# The Effects of Body Positions and Abduction Angles on Shoulder Muscle Activity Patterns during External Rotation Exercises

**DOI:** 10.3390/healthcare11141977

**Published:** 2023-07-08

**Authors:** Jung-Ha Sung, Woosung Jung, Junsig Wang, Jung-Hyun Kim

**Affiliations:** 1 Graduate School of Physical Education, Kyung Hee University, Yongin-si 17104, Republic of Korea; jung.ha@khu.ac.kr (J.-H.S.); jbjjb98@khu.ac.kr (W.J.); junghyun.kim@khu.ac.kr (J.-H.K.); 2 Department of Sports Medicine, Kyung Hee University, Yongin-si 17014, Republic of Korea

**Keywords:** shoulder muscle activity, muscle co-contraction, electromyography, external rotation, rehabilitation

## Abstract

Background: Excess and repetitive glenohumeral external rotation (ER) is at a higher risk for shoulder injuries, which occurs frequently in professional sports and specific occupations. Current rehabilitation programs emphasize selective targeting of muscle activity, which can help restore dysfunctional muscle imbalances or prevent injuries. However, the impact of different body postures on GH muscle activity during ER exercises has not been fully understood. Therefore, the purpose of this study was to investigate shoulder EMG activity for different body positions and humeral abduction angles during ER exercises. Method: Twenty-three healthy young men (age: 22.3 ± 2.3, height: 175.75 ± 4.02, mass: 75.37 ± 9.14) participated in this study. Surface Electromyography was recorded from seven shoulder muscles: upper trapezius, lower trapezius, serratus anterior, infraspinatus, and deltoid. Six ER exercises: three postures (sitting, supine lying, and prone lying) and two abduction angles (ABD; 45° and 90°) were tested using an isokinetic dynamometer. Results: During a sitting position, the lower trapezius/upper trapezius muscle activity ratios were significantly increased for sitting compared to supine lying and prone lying (*p* < 0.001, *p* = 0.004). Serratus anterior/upper trapezius co-contraction indices were significantly increased for 90° than 45° ABD (*p* < 0.001). Conclusion: These findings can provide insight into new training programs aimed at restoring GH muscle imbalances.

## 1. Introduction

The glenohumeral (GH) joint is the ball-and-socket joint that has the greatest mobility but the poorest stability in the human body. Shoulder muscle imbalance is closely associated with potential shoulder injuries such as shoulder dyskinesis, impingement, and adhesive capsulitis [1,2,3,4]. These shoulder disorders are common not only in overhead athletes but also in occupations such as hairdressers, slaughterhouses, mechanics, and painters, which require overhead work, heavy lifting, or forceful tasks [5,6,7,8]. Therefore, a specific and effective resistance training method to balance the shoulder musculature is critical for preventing injury and restoring function.

Shoulder external rotation (ER) exercises have been commonly employed in many rehabilitation programs to restore shoulder muscle function and prevent subsequent shoulder injuries [4,9,10,11,12,13,14,15,16,17]. During shoulder ER exercises, stability of shoulder muscles is needed to enhance shoulder function. Also, many studies have reported abnormal shoulder muscle activity in patients with shoulder injuries such as impingement, rotator cuff disease, adhesive capsulitis, and GH joint instability during ER exercises [1,2,3,4,12,15]. Patients with shoulder impingement and scapular muscle imbalance showed increased upper trapezius and decreased lower trapezius muscle activity [1,2,3,4]. The excessive contribution of deltoids and weakness of infraspinatus may lead to humeral translation and shoulder instability [4,12,15]. Balance of the rotator cuff and surrounding shoulder muscle activity is important to generate proper couple forces and prevent from narrowing of the subacromial space [3]. Thus, ER exercises appear to be a key component of the training program to reduce shoulder pain and improve shoulder function.

**Figure 1:**
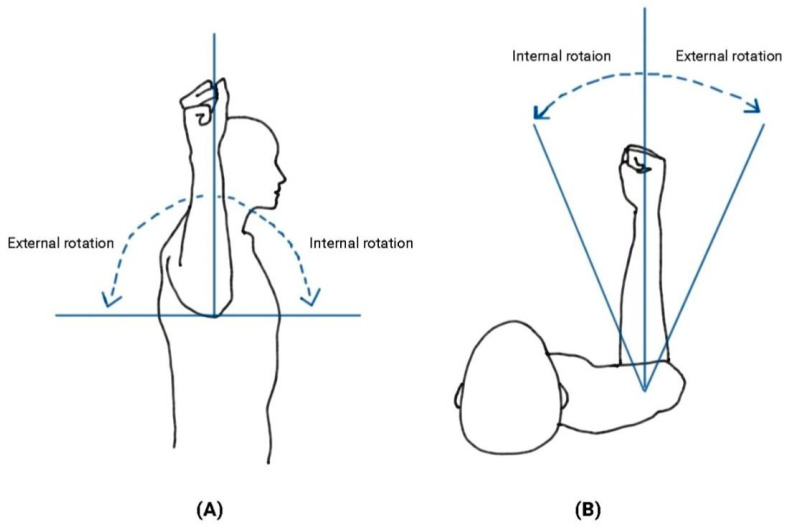
(**A**) External and internal rotation in scapular humeral abduction at 90°. (**B**) External and internal rotation without scapular humeral abduction (at 0°).

During ER exercises, a preferred humeral position and shoulder angle vary according to different body positions (i.e., sitting and standing), which could affect muscle activity patterns of the shoulder musculature. For instance, lower trapezius muscle activity was higher during ER in a prone lying position compared to a standing position [4,16]. Upper trapezius muscle activity decreased during ER at 45° shoulder abduction compared to 90° [4,16]. These findings support the idea that different humeral scapular plane abduction (ABD) angles would alter shoulder muscle activity (Figure 1). However, the effects of different body positions and different shoulder ABD angles on shoulder muscle activity remain largely unknown. An effective ER exercise method will help guide rehabilitation programs aimed at selective muscle-strengthening strategies while decreasing the excessive activation of other shoulder muscles.

Therefore, the purpose of this study was to investigate the effects of different body postures and humeral ABD angles on shoulder EMG activity during ER exercises. We hypothesized that: (1) The ratio of the lower trapezius/upper trapezius EMG activity would be significantly increased for sitting compared to other positions. (2) The co-contraction of the serratus anterior/upper trapezius would be increased for 90° compared to 45° ABD. Previous studies indicated that the upper trapezius and serratus anterior EMG activity was affected by different body positions and humeral ABD angles, which could change muscle tension and the postural role during ER exercises [4,16]. This study will provide a further understanding of appropriate ER exercise methods for developing rehabilitation and injury prevention programs.

## 2. Materials and Methods

### 2.1. Participants and Ethical Approval

A total of 23 healthy young men with a mean (±SD) age of 22.3 (±2.3) years, height of 175.75 (± 4.02) cm, and mass of 75.37 ± 9.14 kg were recruited in this study. G*power was used to calculate a sample size of 23 using our pilot data to determine a minimum estimated effect size of 0.30 with an alpha error probability of 0.05 and a power of 0.80.

Participants were also free of any pain or pathology that would prevent them from being able to perform shoulder external rotation exercises. Prior to participating in the study, each participant read and signed an informed consent form approved by the university’s institutional review board (IRB ID: KHGIRB-21-459).

### 2.2. Experimental Procedure

**Figure 2:**
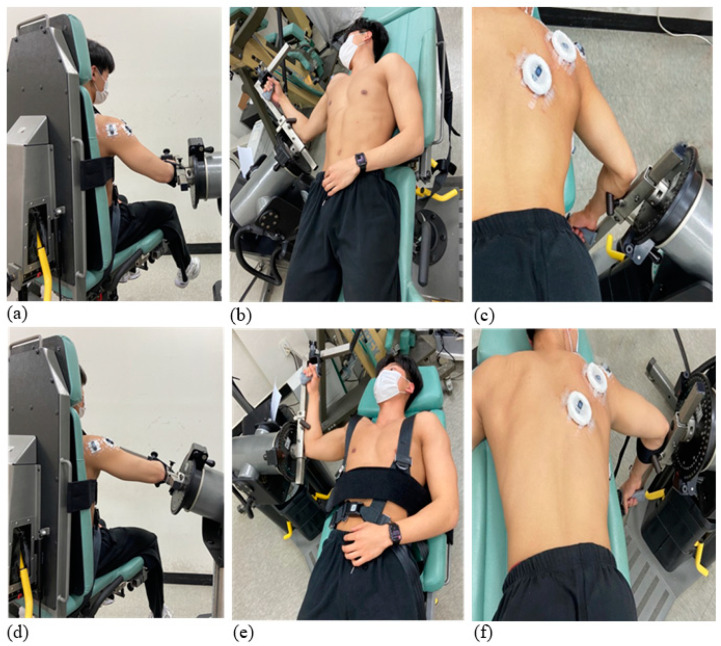
Experimental condition. (**a**) Sitting at 45° abduction, (**b**) supine lying at 45° abduction, (**c**) prone lying at 45° abduction, (**d**) sitting at 90° abduction, (**e**) supine lying at 90° abduction, (**f**) prone lying at 90° abduction.

**Figure 3:**
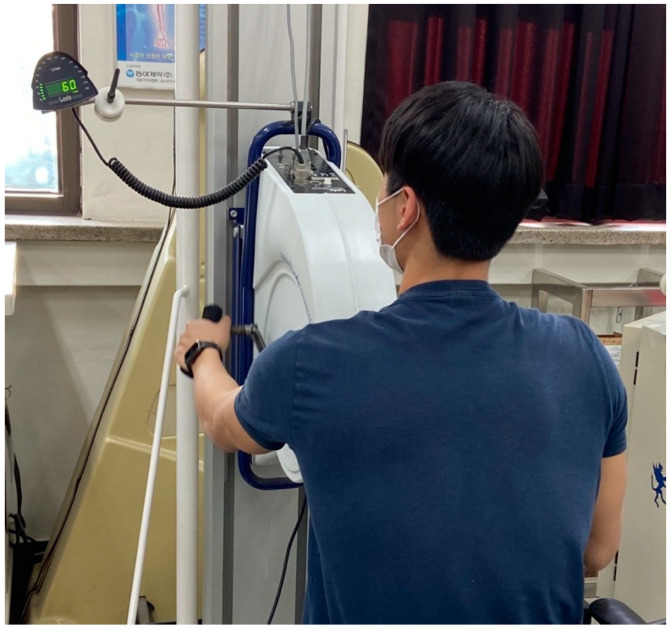
Warm-up exercise using an upper-body ergometer.

**Figure 4:**
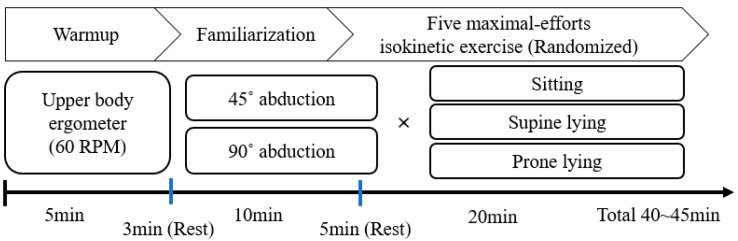
Experimental procedures.

The participants were tested for six ER exercise conditions, which consisted of three positions (sitting, supine lying, and prone lying) and two shoulder ABD angles (45° and 90°; Figure 2). The testing started with a warm-up session using an upper body ergometer for 5 min at 60 revolutions per minute (RPM; Figure 3). After that, an isokinetic dynamometer (Cybex Humac Norm Dynamo meter Model 770, CSMi, Stoughton, MA, USA) was calibrated according to the manufacturer’s recommendations. The participants performed five maximal-effort repetitions at an angular velocity of 60°/s on an isokinetic dynamometer. They completed the six exercise conditions in a random order as practice sessions to familiarize themselves with each ER shoulder exercise (Figure 4). Verbal explanations and instructions were given during the practice trials. Thereafter, the participants were asked to perform the shoulder ER movements under the six conditions at 60°/s and a range of motion between 30° internal rotation and 90° external rotation. The order of the experimental conditions was randomized and, a total of six trials (three positions: sitting, supine lying, and prone lying × two shoulder abduction angles) were performed. The range of motion was set at 120° to avoid any risk of shoulder injuries or excessive joint compressive loads.

During the sitting position, participants were seated with the seatback. The dynamometer was rotated at 5° and inclined to the axis of the humerus aligned with the axis of the dynamometer according to each shoulder abduction angle (45° or 90°). During the supine and prone lying, the dynamometer and chair were adjusted to 45° and 90° shoulder abductions. The dynamometer chair was adjusted to the axis of the acromioclavicular joint relative to the mechanical axis of the dynamometer. Velcro straps and pads were also used to maintain the correct body postures during each ER exercise condition. Each participant was allowed to rest as much as necessary between conditions, with a minimum break of two minutes. All tests were conducted by two researchers experienced in dynameter evaluations including the participant positioning and the hardware and software operations.

### 2.3. Electromyography

Surface EMG (EMG; Cometa Inc, MI, Italy; 2000 Hz) was recorded from seven muscles: upper trapezius (UT), lower trapezius (LT), infraspinatus (ISP), anterior deltoid (AD), middle deltoid (MD), posterior deltoid (PD), serratus anterior (SA) during each condition. The skin was prepared by shaving any excess hair and using an abrasive paste (Idopharm; Ido Skin Swab, Ansan, Gyeonggi-do, Republic of Korea). Electrodes were placed parallel to muscle fibers according to standard SENIAM guidelines [18], and all attachments were conducted by one researcher (JHS) for consistency (Table 1). Raw EMG signals were evaluated for corrupted data using visual amplitude inspection and power spectral analysis [19]. The raw EMG signals were band-pass filtered using a 4th-order Butterworth filter between 10 and 450 Hz, to reduce contamination from movement artifacts. In addition, EMG waveforms were notch-filtered at 60 Hz with a 4th-order Butterworth filter to eliminate the effects of signal interference from nearby electronic sources. The data were then rectified and filtered using a low-pass filter at 10 Hz to obtain the EMG linear envelope. Individual muscle EMG amplitudes were calculated as the average linear envelope during the ER exercises, then normalized to the peak amplitude for the baseline condition (45° ABD while sitting). Co-contraction indices were calculated using the Equation below [20]:

$$CCI_{m1:m2}=ave\left\{\overset{i=final}{\underset{i=initial}{\sum }}\frac{min\left\{EMG_{m1}\left(i\right),\;EMG_{m2}\left(i\right)\right\}}{max\left\{EMG_{m1}\left(i\right),\;EMG_{m2}\left(i\right)\right\}}\left(EMG_{m1}\left(i\right)+EMG_{m2}\left(i\right)\right)\right\}$$

In this equation, the variables m1/m2 represent the two specific muscles under analysis. The initial and final were defined as 1–100% of ER movement. The term “min” corresponds to the EMG linear envelope values associated with the less active muscle group, while “max” represents the EMG linear envelope values of the more active muscle group at each time point. Consequently, the Co-contraction Index (CCI) was calculated during shoulder ER exercises. CCIs were calculated for (m1:m2): UT:LT, SA:UT, and ISP:PD. In addition, EMG activity ratios of LT/UT, SA/UT, and ISP/PD were calculated using mean EMG values during the ER movement. All data analysis was conducted using the custom MATLAB code.

### 2.4. Statistical Analysis

All statistical analysis was performed using R version 4.1.3 (Rstudio, Boston, MA, USA) with a significance level of 0.05. The effect of six ER exercise conditions on shoulder muscle EMG was tested with repeated measures analyses of variance (ANOVA). The two-factor repeated measures ANOVAs were used to test the effect of the position (sitting, supine lying, and prone lying) and shoulder abduction angle (45° vs. 90°). Bonferroni post hoc tests were used for multiple comparisons when the main effect of position (sitting vs. supine lying vs. prone lying) was significant.

## 3. Results

Mean EMG amplitudes, ratios, and co-contraction indices of shoulder muscles during ER exercises are described in Table 2 and Table 3.

### 3.1. EMG Activity Amplitudes

ANOVA indicated significant main effects of position on AD (*p* = 0.009), MD (*p* = 0.013), ISP (*p* < 0.001), UT (*p* < 0.001), and SA (*p* = 0.005) muscle activity amplitudes (Table 2). AD, MD, and UT muscle activity increased (*p* = 0.005, *p* = 0.008, *p* = 0.005) while SA muscle activity decreased (*p* = 0.02) when comparing prone lying to sitting. MD muscle activity increased for supine lying compared to sitting (*p* = 0.001). Additionally, ISP and UT muscle activity also significantly increased for prone lying compared to supine lying (*p* < 0.001, *p* < 0.001). ISP muscle activity increased for sitting compared to supine lying (*p* < 0.001). Significant main effects of shoulder ABD angles were found for UT and SA muscle activity (*p* < 0.001, *p* < 0.001). The UT and SA muscle activity was higher for 90° than 45° ABD (*p* < 0.001, *p* = 0.022; Table 2).

**Figure 5:**
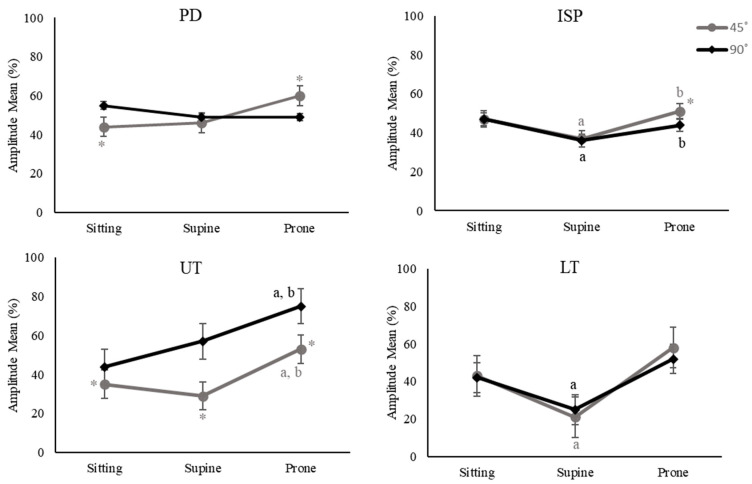
Results of simple effects for PD, posterior deltoid; ISP, infraspinatus; UT, upper trapezius; LT, lower trapezius expressed as a percentage of maximum voluntary contraction (MVC) (amplitude mean %) across 3 positions and 2 abduction (ABD) angles for shoulder external rotation (ER) exercises. (^a^ *p* < 0.05 vs. sitting, ^b^ *p* < 0.05 vs. supine lying, * *p* < 0.05 vs. 90° ABD).

Significant interaction effects of position and ABD angles were found on PD, ISP, UT, and LT muscle activity. Therefore, simple effects for each combination of position and ABD angle were tested. For example, PD muscle activity was higher for 90° ABD than 45° in sitting (*p* < 0.001), while higher for 45° ABD than 90° in pone lying (*p* = 0.038; Figure 5). ISP muscle activity was higher for prone lying and sitting than supine lying for both 45° and 90° ABD (*p* < 0.001, *p* = 0.005, *p* = 0.014, *p* = 0.002; Figure 5), thus supporting that the main effects were true for ISP; however, ISP muscle activity was higher for 45° ABD than 90° only for prone lying (*p* = 0.03; Figure 5). UT muscle activity was higher for prone lying than sitting and supine lying for both 45° and 90° ABD (*p* = 0.011, *p* = 0.001, *p* < 0.001, *p* < 0.001; Figure 5), which indicated that the main effects were true for UT. Furthermore, LT muscle activity was higher for sitting than supine lying for both 45° and 90° ABD (*p* < 0.001, *p* < 0.001; Figure 5).

### 3.2. Ratios of Shoulder Muscles

Univariate ANOVA revealed significant main effects of position on LT/UT and SA/UT ratios (*p* < 0.001, *p* < 0.001). The LT/UT ratio decreased for supine lying and prone lying compared to sitting (*p* < 0.001, *p* = 0.004). SA/UT ratios also decreased for prone lying compared to sitting and supine lying (*p* < 0.001, *p* = 0.017). A significant main effect of shoulder angle was found on LT/UT ratios. LT/UT ratios were higher for 45° than 90° ABD (*p* < 0.001; Table 3).

### 3.3. Co-Contraction Indices of Shoulder Muscles

**Figure 6:**
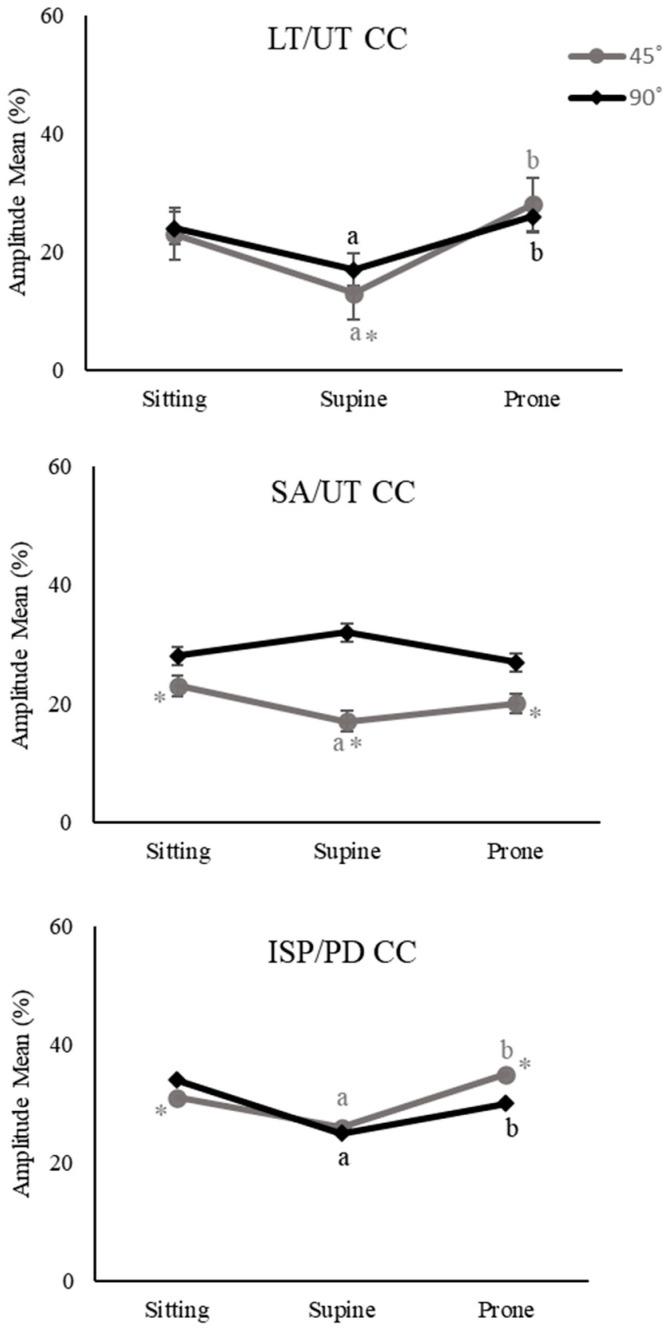
Results of simple effects for co-contraction (CC) of LT/UT, lower trapezius/upper trapezius; SA/UT, serratus anterior/upper trapezius; ISP/PD, infraspinatus/posterior deltoid expressed as a percentage of maximum voluntary contraction (MVC) (amplitude mean %) across 3 positions and 2 abduction (ABD) angles for shoulder external rotation (ER) exercises. (^a^ *p* < 0.05 vs. sitting, ^b^ *p* < 0.05 vs. supine lying, * *p* < 0.05 vs. 90° ABD).

Significant interaction effects were found for LT/UC CC, SA/UT CC, and ISP/PD CC indices. Thus, follow-up tests were performed and simple effects for each combination of position and shoulder angle were tested. LT/UT CC indices were higher for sitting and prone lying than supine lying for both 45° and 90° ABD (*p* < 0.001, *p* < 0.001, *p* = 0.016, *p* = 0.053; Figure 6), indicating that the main effect of the position was true. But, LT/UT CC indices were higher for 90° than 45° ABD only for supine lying (*p* = 0.036; Figure 6). In addition, SA/UT CC indices were higher for sitting than supine lying for 45° ABD (*p* = 0.048; Figure 6). When comparing 45°, SA/UT CC indices were higher for 90° across sitting, supine, and prone lying (*p* = 0.017, *p* < 0.001, *p* = 0.01; Figure 6). ISP/PD CC indices were higher for sitting and prone lying than supine lying for both 45° and 90° ABD (*p* = 0.017, *p* = 0.002, *p* < 0.001, *p* = 0.034; Figure 6). When comparing 45° to 90° ABD, ISP/PD CC decreased for sitting but increased for prone lying (*p* = 0.026, *p* = 0.008; Figure 6).

## 4. Discussion

The purpose of this study was to investigate the effects of different body postures and humeral ABD angles on shoulder EMG activity during ER exercises. An optimal shoulder ER exercise strategy to target the weakened muscles and reduce overactivated muscles is of value to the current rehabilitation programs for regaining muscle balance and enhancing functional mobility.

### 4.1. Trapezius

As expected, UT muscle activity increased for 90° compared to 45° ABD. In addition, UT muscle activity increased for prone lying compared to sitting and supine lying. Previous studies found that patients with shoulder impingement or scapular muscle imbalance attenuated activity in the LT muscle and hyperactivated activity in the UT muscle [1,2,3,21,22,23]. In patients with shoulder impingement or dysfunction, increased UT muscle activity affects the excessive elevation of the clavicle and scapula [3]. Thus, it should be noted that ER in prone lying is avoided for patients with shoulder instability. For LT muscle, a previous study reported increased LT activity for prone lying compared to standing position, which may be due to motion alignment and increased tension in muscle fibers [4,16]. In this study, a higher LT activity was found for sitting compared to supine lying, which may indicate that the sitting ER exercise could improve LT muscle strength. Along with the results of the UT and LT muscles, the LT/UT ratio increased for sitting compared to supine and prone lying. These findings may be explained by minimized tension and the postural role of the UT muscle during sitting ER exercises [4]. In addition, LT/UT ratios increased to 45° compared to 90° ABD, indicating less dominant activation of UT for 45° ABD. Therefore, these EMG changes suggest that sitting ER at 45° ABD may be beneficial for patients with shoulder problems to reduce joint compression until they reach a sufficient level of functional recovery.

### 4.2. Serratus Anterior

Sufficient activation of SA helps generate a proper force couple with the trapezius muscle and lead to normal scapulohumeral rhythm [4,21,24]. Appropriate SA muscle activity to a relevant level is important for scapular stabilization and shoulder function during ER exercises [25]. We found that SA muscle activity increased in sitting compared to prone lying and SA/UT ratios decreased for prone lying compared to sitting and supine lying. These findings suggest that both sitting and supine lying ER exercises are good methods for strengthening the SA muscle and improving the stabilization of the scapulothoracic region. Previous studies reported that SA muscle activity increased for 90° compared to 0° ABD during standing ER exercises [4,14]. It is also found that SA activity tends to increase with humeral elevation [14]. As with increased SA and UT muscle activity, SA/UT CC indices were also increased for 90° compared to 45° ABD. Given that the ER at 90° ABD increases co-contraction of SA/UT, it may be more beneficial for healthy individuals who are focused on improving their performance rather than correcting shoulder muscle imbalances [3]. However, for patients with shoulder imbalances who need to restore their muscular function rather than enhance their performance, the ER at 90° ABD is of concern when considering increased co-contraction of SA/UT [26]. As mentioned before, overactivation of the UT and SA could lead to an excessive elevation and an anterior tilt of the scapula [3,4,21]. Additionally, these changes for ER at 90° ABD may put patients with shoulder problems at risk for compressive loads and the narrowed subacromial space [1,3,27,28]. Thus, we recommend ER exercises at a low ABD angle for patients with shoulder problems who need to recover their abnormal muscle activation patterns.

### 4.3. Deltoids

Excessive AD and MD muscle activity is linked to a higher risk of superior humeral head translation, which causes subacromial impingement by narrowing the coracoacromial arch [4,12,14,15]. In addition, unnecessary PD activity interferes with selective ISP strengthening and causes compensatory mechanisms [12,13,15]. We found that AD and MD activities decreased for sitting compared to prone lying during ER exercises. In addition, MD muscle activity decreased for sitting compared to supine and prone lying. One of the potential reasons for the results is motion alignment and muscle tensions at the GH joint during supine and prone lying. A previous study has shown that the acromiohumeral distance on radiographs increases for the standing position compared to supine lying [29]. Likewise, greater muscle tension due to reduced acromiohumeral distance may alter AD and MD activities. Furthermore, this could be the reason why patients with shoulder disorders often experience shoulder pain and sleep disturbances during lying positions [30,31,32]. Interestingly, we also found that PD muscle activity increased for 90° ABD than 45° in a sitting position. Previous studies also recommended ER exercises at 0° ABD that reduced PD activity than 90° ABD [11,15]. Thus, our findings suggest that less than 90° ABD angle helps decrease excessive PD muscle activity during sitting ER exercises.

### 4.4. Infraspinatus

ISP plays an important role in reducing the risk of superior shoulder joint distraction [4,12,14,15]. ISP muscle activity increased for sitting and prone lying compared to supine lying in this study. A previous study reported increased ISP activity for prone lying compared to sitting, which is different from our results (no significant changes) [15]. The reason may be due to the different measurement methods used in this study. We collected data during isotonic contractions, whereas the previous study measured the EMG activity during isometric contractions at the end of ER exercises. It is believed that muscle recruitment thresholds can be affected by the type of muscle contraction. The motor neuron pools that innervate the muscle fibers have different activity distributions for isometric and isotonic movements [33]. Moreover, the participants in this study were seated with a seatback, and the trunk was fixed with Velcro straps, but it is unclear whether the seatback and Velcro straps were used in the previous study. Using the seatback and strap may help maintain proper body postures and stability of the shoulder blades during ER exercises, which could affect the EMG activity.

Different ABD shoulder angles did not affect ISP muscle activity for sitting and supine lying during ER exercises. Previous studies reported that ER exercises at low ABD (0–45°) have an increased tendency to target the ISP activity compared to ≥90° ABD, [11,12,34,35] except for one study [4] Another study also found no differences in ISP muscle activity between ER with and without adduction [13] We found that ISP muscle activity increased for 45° compared to 90° ABD only for prone lying, indicating that ISP activity is sensitive to different ABD angles only for prone lying. For that reason, during the prone lying ER, muscle tension at 45° ABD may make ISP muscle a more effective external rotator depending on the length–tension relationship. Additionally, a recent cadaver study demonstrated that the moment arm lengths of the ISP muscles vary depending on superior, middle, and inferior fibers during scapular plane elevation [36]. Overall, ER at lower ABD angles seems to be more beneficial for targeting ISP without excessive recruitment of PD compared to higher ABD angles.

There are several limitations of this study. First, the increased co-contraction values do not always indicate a positive effect, so further studies should provide co-contraction values to judge ‘good’ and ‘bad’ values for patients with shoulder problems. Second, we only focused on superficial shoulder muscles; therefore, it is not clear how different positions and ABD shoulder angles impact other rotator cuff muscles such as subscapularis, supraspinatus, and teres minor during ER exercises. Third, the sample size was small (*n* = 23) and thus our results may have limited generalizability. Lastly, only five trials of ER were examined, and analyses of more trials may produce more reliable results.

## 5. Conclusions

In summary, our findings suggest that a sitting position at 45° ABD is effective in selectively strengthening the LT muscle, as evidenced by an increased ratio of the LT/UT and decreased AD and MD activity. To target the SA muscle, the prone lying ER is not recommended because of the deceased SA/UT ratio. For healthy individuals, ER at 90° ABD may have some benefits since this could improve shoulder muscle cooperation stability with increased SA/UT co-contraction indices. For patients with shoulder injuries, however, ER at 45° ABD is likely to be more beneficial for restoring SA function. This study may provide valuable information for developing shoulder rehabilitation programs when considering shoulder muscle imbalances and individualized targeting of external rotator exercises.

## Figures and Tables

**Table 1:** Location of surface electromyography (EMG) sensor.

Muscle	Electrode Placement
Upper trapezius (UT)	Midway between the line from the acromion and the spine on vertebra C7.
Lower trapezius (LT)	2/3 on the line from the trigonal spinae to the 8th thoracic vertebra.
Infraspinatus (ISP)	Center of the infraspinous fossa
Anterior deltoid (AD)	One finger width distal and anterior to the acromion
Middle deltoid (MD)	Greatest muscle belly which placed from the acromion to the lateral epicondyle of the elbow.
Posterior deltoid (PD)	2 cm below the posterior angle of the acromion
Serratus anterior (SA)	Over the 7th rib in the anterior axillary line

**Table 2:** Normalized EMG amplitudes during different ER exercises.

	Sitting	Supine	Prone	45°	90°	Position	Angle	Position × Angle
Anterior deltoid (AD)	28 (0.14)	35 (0.23)	52 ^a^ (0.53)	36 (0.31)	41 (0.39)	*p* = 0.009	*p* = 0.36	*p* = 0.58
Middle deltoid (MD)	49 (0.21)	61 ^a^ (0.19)	79 ^a^ (0.7)	58 (0.39)	68 (0.5)	*p* = 0.013	*p* = 0.11	*p* = 0.42
Posterior deltoid (PD)	50 (0.14)	48 (0.19)	55 (0.29)	50 (0.24)	51 (0.19)	*p* = 0.31	*p* = 0.58	*p* = 0.002
Infraspinatus (ISP)	47 ^b^ (0.1)	37 (0.1)	48 ^b^ (0.15)	45 (0.14)	43 (0.12)	*p* < 0.001	*p* = 0.11	*p* = 0.03
Upper trapezius (UT)	39 (0.17)	43 (0.23)	64 ^a,b^ (0.29)	39 * (0.21)	58 (0.26)	*p* < 0.001	*p* < 0.001	*p* = 0.01
Lower trapezius (LT)	42 (0.09)	23 (0.12)	55 (0.95)	41 (0.62)	39 (0.52)	*p* = 0.15	*p* = 0.56	*p* = 0.03
Serratus anterior (SA)	53 (0.45)	46 (0.38)	33 ^a^ (0.17)	37 * (0.3)	51 (0.4)	*p* = 0.005	*p* < 0.001	*p*= 0.57

Values are % mean (standard deviation). ^a^ *p* < 0.05 vs. sitting, ^b^ *p* < 0.05 vs. supine lying, * *p* < 0.05 vs. 90° ABD.

**Table 3:** EMG activity ratios and co-contraction indices during different ER exercises.

	Sitting	Supine	Prone	45°	90°	Position	Angle	Position × Angle
Lower trapezius/Upper trapezius (LT/UT) ratio	1.27 (0.56)	0.67 ^a^ (0.48)	0.79 ^a^ (0.73)	1.03 * (0.59)	0.79 (0.68)	*p* < 0.001	*p* < 0.001	*p* = 0.31
Serratus anterior/Upper trapezisus (SA/UT) ratio	1.44 (0.84)	1.4 (1.84)	0.57 ^a,b^ (0.29)	1.21 (1.55)	1.05 (0.83)	*p* < 0.001	*p* = 0.34	*p* = 0.09
Infraspinatus/Posterior deltoid (ISP/PD) ratio	1.01 (0.35)	0.86 (0.34)	0.97 (0.33)	0.97 (0.32)	0.92 (0.36)	*p* = 0.13	*p* = 0.1	*p* = 0.06
Lower trapezius/Upper trapezius (LT/UT) co-contraction	0.24 ^b^ (0.07)	0.15 (0.08)	0.27 ^b^ (0.15)	0.21 (0.12)	0.22 (0.12)	*p* < 0.001	*p* = 0.32	*p* = 0.006
Serratus anterior/Upper trapezius (SA/UT) co-contraction	0.26 (0.1)	0.25 (0.13)	0.23 (0.13)	0.2 * (0.09)	0.29 (0.13)	*p* = 0.5	*p* < 0.001	*p* = 0.003
Infraspinatus/Posterior deltoid (ISP/PD) co-contraction	0.32 ^b^ (0.07)	0.26 (0.07)	0.33 ^b^ (0.1)	0.31 (0.09)	0.3 (0.08)	*p* < 0.001	*p* = 0.21	*p* < 0.001

^a^ *p* < 0.05 vs. sitting, ^b^ *p* < 0.05 vs. supine lying, * *p* < 0.05 vs. 90° ABD.

## Data Availability

The data presented in this study are available on request from the corresponding author.
